# Dynamic Covalent Dextran Hydrogels as Injectable, Self-Adjuvating Peptide Vaccine Depots

**DOI:** 10.1021/acschembio.2c00938

**Published:** 2023-02-17

**Authors:** Bowen Fan, Diana Torres García, Marziye Salehi, Matthew J. Webber, Sander I. van Kasteren, Rienk Eelkema

**Affiliations:** † Department of Chemical Engineering, Delft University of Technology, Van der Maasweg 9, 2629 HZ Delft, The Netherlands; ‡ Department of Chemical and Biomolecular Engineering, University of Notre Dame, Notre Dame, Indiana 46556, United States; § Division of Bio-Organic Synthesis, Leiden Institute of Chemistry and Institute of Chemical Immunology, Leiden University, Gorlaeus Laboratory, Einsteinweg 55, 2333 CC Leiden, The Netherlands

## Abstract

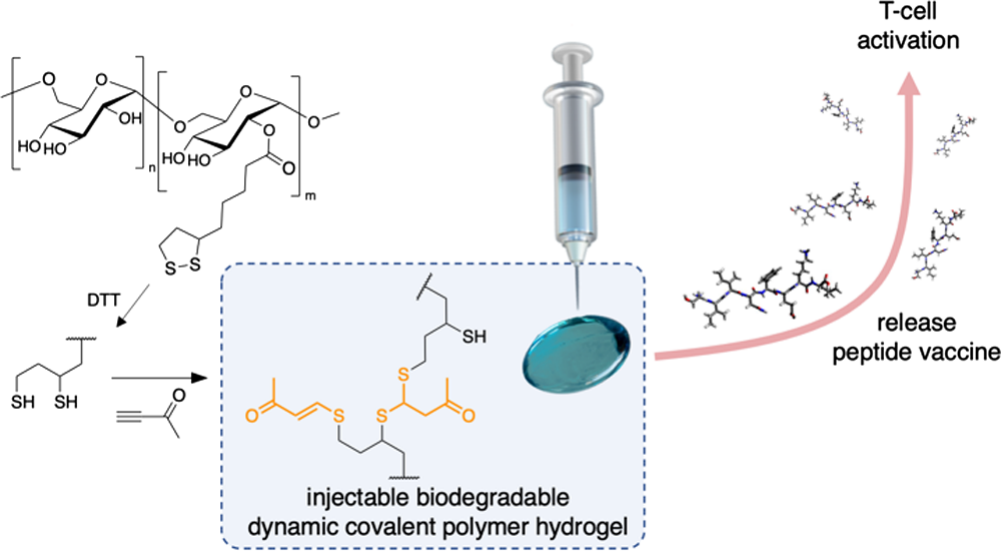

Dextran-based hydrogels are promising therapeutic materials for drug delivery, tissue regeneration devices, and cell therapy vectors, due to their high biocompatibility, along with their ability to protect and release active therapeutic agents. This report describes the synthesis, characterization, and application of a new dynamic covalent dextran hydrogel as an injectable depot for peptide vaccines. Dynamic covalent crosslinks based on double Michael addition of thiols to alkynones impart the dextran hydrogel with shear-thinning and self-healing capabilities, enabling hydrogel injection. These injectable, non-toxic hydrogels show adjuvant potential and have predictable sub-millimolar loading and release of the peptide antigen SIINFEKL, which after its release is able to activate T-cells, demonstrating that the hydrogels deliver peptides without modifying their immunogenicity. This work demonstrates the potential of dynamic covalent dextran hydrogels as a sustained-release material for the delivery of peptide vaccines.

## Introduction

The success of vaccines is strongly dependent on the kinetics of antigen exposure and the subsequent cellular and humoral immune responses induced.^1^ Sustained antigenic exposure within tightly controlled release conditions is therefore sought after to prompt a durable and protective immune response.^1−3^ Sustained-release technologies such as nanoparticles, cationic lipids/liposomes, polysaccharides, and poly(lactic-*co*-glycolic acid) (PLGA) particles can provide these essential spatial and temporal interactions.^3−5^ Nevertheless, these systems must overcome some challenges such as tedious synthesis procedures, low drug-loading capacity, or inability to deliver cargo. As for the cargo, the loaded antigens could face degradation and immunogenicity loss induced by changes in pH, temperature, oxidation/reduction reactions, or other chemical modifications.^6^ Thus, there is an urgent need for delivery systems capable of releasing unmodified antigens. In this regard, an excellent alternative for such systems can be found in hydrogels as they can possess high biocompatibility as well as high loading capacity.^7,8^ Their hydrophilic nature enables the absorption of large amounts of the fluid and bioactive cargo.

The use of hydrophilic polymer scaffolds ensures a low cellular and protein adherence to the gel interface, making them biocompatible.^6,9^ Viscoelastic properties and in vivo degradation characteristics can be tuned through molecular design.^10,11^ The chemical and mechanical properties of hydrogels determine the release kinetics of the cargo, which can be controlled by altering the polymer structure, the density, and type of crosslinker forming the hydrogel, as well as its degradation kinetics.^12−14^ Hydrogels can act as depots for the sustained release of antigens.^15−17^ These depot gels can be introduced in the body by grafting to the skin, surgical implantation, and through injection. Injection requires shear-thinning and self-healing properties that are not observed for permanently crosslinked polymer hydrogels but can be introduced through the use of reversible crosslinks. In this context, Appel et al. described a cellulose-derived hydrogel containing hydrophobic non-covalent crosslinks for the sustained release of model protein antigens.^15^

In an effort to develop injectable hydrogel antigen depots that do not use hydrophobic interactions, we were interested to evaluate the use of dextran polymers crosslinked through dynamic covalent bonds for the release of peptide antigens. Peptide vaccines have been at the forefront in the recent spate of “molecularly defined” anti-cancer vaccines.^18,19^ However, to date, they have precluded hydrogel-based delivery, with only large protein antigens having been delivered in injectable hydrogels. The small size and poor solubility of many antigenic peptides mean that delivering these agents presents additional restraints on the carrier materials.

Here, we report the synthesis, characterization, and mechanical properties of an injectable dextran hydrogel containing dynamic covalent crosslinks and evaluation of prolonged T-cell activation by the release of peptide antigens. The biocompatibility of dextran has been previously reported making this polymeric hydrogel a very suitable system for the controlled release of antigens.^20−22^ As a dynamic covalent crosslink, we use the double Michael addition of thiols to alkynones, affording a reversible dithiane link that will slowly degrade in the biological environment. We demonstrate the feasibility of this novel dextran hydrogel as a delivery system by loading it with the SIINFEKL peptide, a minimal CD8-restricted T-cell epitope peptide. Moreover, this peptide was successfully released without losing its immunogenicity and it was taken up and processed by dendritic cells, resulting in its efficient presentation in the context of MHC class I molecules and the subsequent antigen-specific T-cell activation.

We have recently reported a polymer hydrogel that is crosslinked through thiol-alkynone Michael addition dynamic covalent chemistry.^23^ The gels were made from tetra-thiol PEG star polymers that react with a small-molecule alkynone to form a β-dithiane carbonyl dynamic crosslink.^24−26^ Resulting from the presence of this dynamic covalent crosslink, these polymer gels are shear thinning and self-heal after the stress is removed. Because of these properties, the gels can be injected using a syringe, where they form stable gel particles immediately upon exiting the needle. Combining injectability with high water content and crosslinks that show dynamic behavior under physiological conditions makes these materials interesting candidates for evaluation as injectable antigen depot vehicles for vaccination.

**Figure 1:**
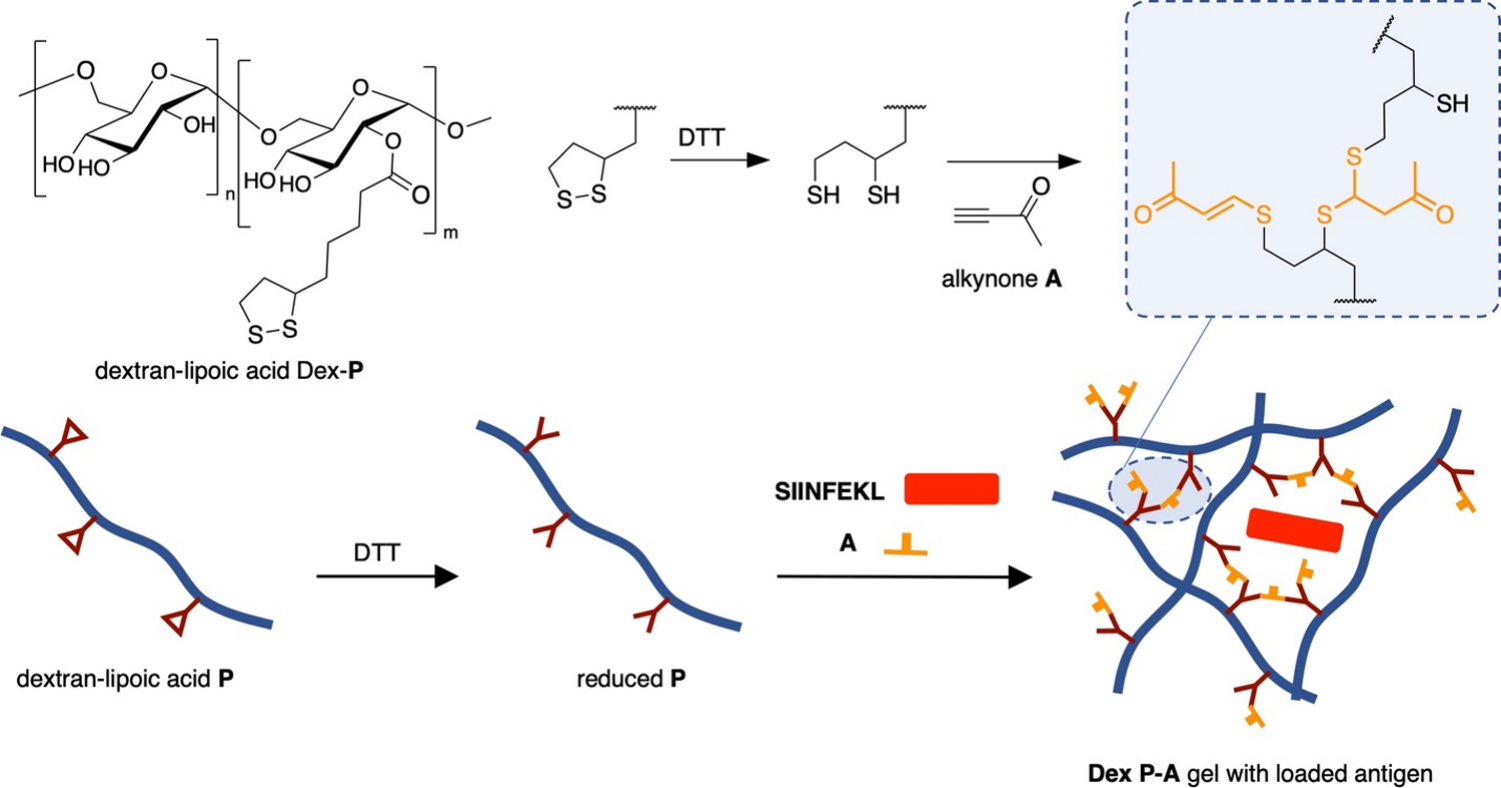
General concept and materials. Thiols formed by the reduction of LA side chains on dextran polymer Dex-**P** react with alkynone **A** to form the single and double Michael adducts. **A**-crosslinked polymer gels (Dex **P-A**) can be loaded with peptide antigens such as SIINFEKL and used for T-cell activation.

The original research employed a tetrathiol PEG star polymer as the polymer backbone of the gel. Such star polymers have limited availability and are difficult to functionalize further. Moreover, the free thiol groups may form disulfides through oxidation, leading to undesired crosslinking. Building on this work, we opted to develop a dextran-based polymer with side-chain-grafted lipoic acid (LA) as a masked dithiol. Reduction of the LA disulfide leads to the formation of two thiol functionalities, which mostly revert to the ring-closed disulfide upon oxidation, thereby avoiding undesired crosslinking. Using a graft copolymer instead of an end-functionalized star polymer allows control over the crosslink-to-polymer ratio. After reduction, the free thiols on the LA-functionalized dextran **P** can react with alkynone **A** to form a polymer network crosslinked with dynamic covalent double Michael addition products (Figure 1).

## Results and Discussion

### Hydrogel Synthesis

**Figure 2:**
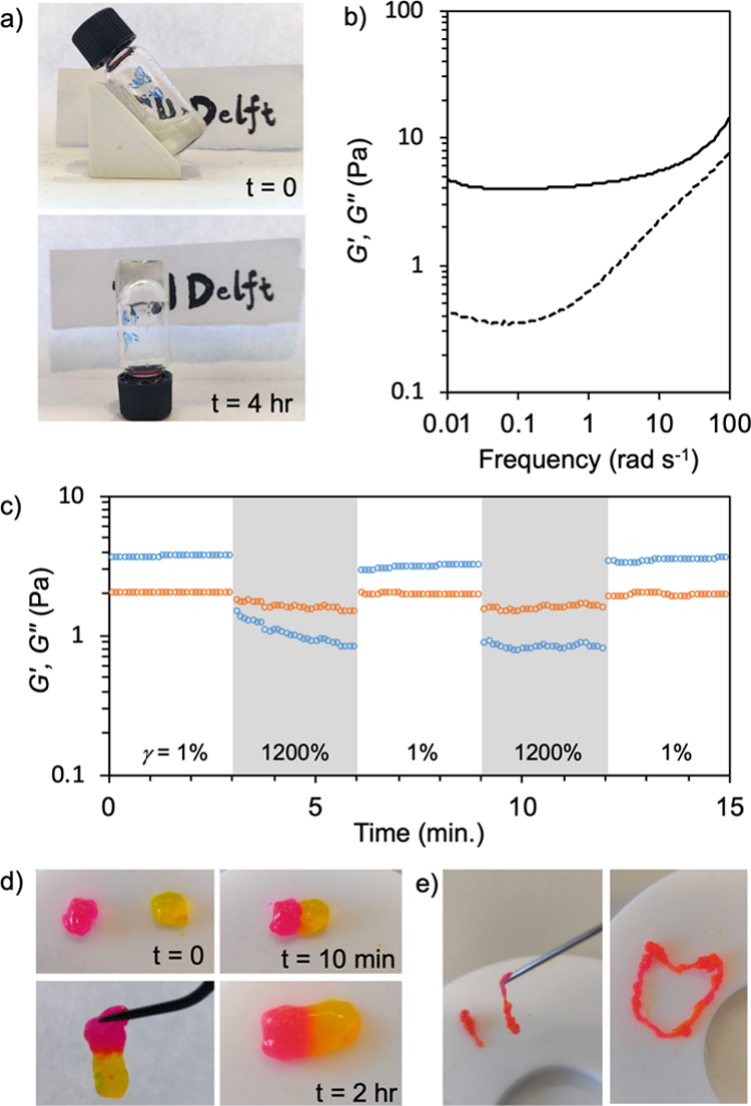
Dex **P-A** hydrogel formation and its mechanical properties, self-healing, and injectability. (a) Hydrogel formation by mixing **P70-4.2**, DTT, and alkynone **A** PB8.2 solutions sequentially: after 10 min of mixing **P70-4.2** and DTT solutions, **A** in PB8.2 was added into the mixture, initiating the gelling process (top); after 4 h of reaction, a slightly turbid hydrogel formed, which could hold its own weight when inverted (bottom). (b) Rheological frequency sweep of the hydrogel 6 h after mixing, showing that *G*′ (solid line) is higher than *G*″ (dashed line) over the entire frequency range (strain (γ) = 0.5%, frequency = 100–0.01 rad s^–1^, 25 °C). (c) Continuous step-strain measurement of the hydrogel; the strain is switched from 1 to 1200% for two cycles. (d) Macroscopic self-healing of the hydrogel. Two cube-shaped gels (4 × 10 × 10 mm) were colored red and yellow using rhodamine B and fluorescein, respectively (top left). The two gels were pressed together and found to have connected after 10 min (top right). The rejoined gel can be lifted using a tweezer (bottom left). After 2 h, the interface between the two gels had disappeared, and the dyes could diffuse over the interface (zoom, bottom right). (e) A 0.6 ± 0.2 mm strip-shaped hydrogel formed after hand-pressed extrusion through a 20G syringe needle (left). Rhodamine B was added to the gels for visualization. The extruded structures could hold their shape over extended periods (right).

To make the hydrogel vaccine, we first synthesized LA-functionalized dextran (Dex-**P*x*-*y***, with *x* indicating the dextran molecular weight and *y* indicating the degree of functionalization with LA). We synthesized several versions of Dex-**P** using two methods: dextran was either reacted with LA anhydride catalyzed by dimethylaminopyridine (DMAP) or with LA catalyzed by DPTS (the 4-toluenesulfonate salt of DMAP). Starting from dextran with *M*_w_ = 20, 70, and 500 kDa, we synthesized Dex-**P** polymers with degrees of substitution between 3.1 and 10.5 (Table 1). Dex-**P** polymers with a high degree of substitution were not sufficiently soluble in an aqueous solvent and were not investigated further. We next determined whether the polymers could form gels upon reaction with alkynone **A** in sodium phosphate buffer (100 mM phosphate, pH = 8.2, “PB8.2”). For the dithiothreitol (DTT)-mediated reduction of native LA, we determined that around 67% disulfide is reduced in 10 min using 1 equiv of DTT in PB8.2 (Figure S6). Based on this result, we treated a polymer solution for 10 min with DTT (1 equiv with respect to the dithiolane ring content of the polymer) to reduce the LA side-chain disulfides. After DTT reduction, we added **A** (1 equiv with respect to DTT), and the mixtures were left to react for 4 h at room temperature to allow crosslink formation. Of the soluble Dex-**P** polymers, only the 70 kDa dextran with DS = 4.2 (**P70-4.2**), made using the anhydride method, showed gelation. This polymer formed a turbid hydrogel after activation with DTT and subsequent reaction with **A** (10 wt % polymer in PB8.2) (Figure 2a). **P70-4.2-d** synthesized by the DPTS catalysis method does not form a gel. This effect may be caused by different substitution patterns of dextran depending on the use of the anhydride method or DPTS. For **P70-4.2**, the ^1^H NMR spectrum shows that LA esterification takes place mostly at the C2 hydroxyls of dextran (Figure S2). For **P70-4.2-d**, however, a new peak at 5.27 ppm in the ^1^H NMR spectrum suggests substantial esterification at both C3 and C2 hydroxyls (Figure S5).^27^

**Table 1:** Synthesis, Solubility, and Gelation of Dex-**P** with Varying Molecular Weight and Degree of Substitution

Dex-**P**	dextran *M*_w_ (kDa)	molar ratio of LA to AHG^a^	degree of substitution^b^	**P** solubility^c^	gelation^e^
P20-6.4	20	0.5	6.4	soluble	liquid^d^
P70-3.1	70	0.3	3.1	soluble	liquid^d^
**P70-4.2**	**70**	**0.5**	**4.2**	**soluble**	**gel**
P70-5.9	70	0.8	5.9	low solubility	
P70-4.2-d^f^	70	0.4	4.2	soluble	liquid^d^
P70-6.8-d^f^	70	0.6	6.8	low solubility	
P70-10.5-d^f^	70	0.7	10.5	low solubility	
P500-4.8	500	0.5	4.8	low solubility	

a Molar feeding ratio of LA or LA anhydride to AHG of dextran during the synthesis of Dex **P-A**.

b The degree of substitution is defined as the number of attached LA units per 100 AHG units of dextran and calculated by ^1^H NMR according to the protons of the attached LA group at 3.10 ppm and the dextran glucosidic protons at 4.85 and 5.19 ppm.

c In PB8.2 (20 mg in 180 μL buffer). Low solubility means that even stirred overnight or processed with ultrasonication for 1 h does not lead to complete solubilization.

d The sample still shows flow after 1 day of reaction time, checked by the vial-inversion method.

e Hydrogel formation (10 wt % polymer in PB8.2) was checked by the vial-inversion method. Gel formation means the sample shows no flow within 1 min after inversion.

f Synthesized from dextran-70k and LA catalyzed by DPTS.

All other polymers gave liquid solutions after incubation with **A**. The solubility and gelation results suggest that there is a fine balance between having enough LA groups per polymer chain to allow sufficient crosslink formation and having too many hydrophobic LA groups hindering solubility.

### Viscoeleastic Properties

We investigated the process of hydrogel formation and the mechanical properties of the formed gels by rheological measurements. After reacting for 10 min with DTT, we added **A** to the solution of activated **P70-4.2**, and the mixture was transferred on the rheometer. A rheological time sweep shows a fast gelation, indicated by the crossover of storage modulus (*G*′) and loss modulus (*G*″) after 5 min. After 6 h of reaction, *G*′ approached an equilibrium value (*G*′ = 5.0 Pa and tan δ (*G*″/*G*′) = 0.34) (Figure S7a). A frequency sweep demonstrated that the hydrogel maintains a solid-like state in the range of 100 to 0.01 rad s^–1^ (Figure 2b).

The critical strain (γ) needed to induce a gel–sol transition was determined by a strain sweep from 1 to 1200% (Figure S7c), showing a crossover point of *G*′ and *G*″ at around 1000%. We then examined the ability to self-heal by a continuous step-strain sweep using a cyclic 1 to 1200% strain program (Figure 2c). Upon applying a 1200% strain to the hydrogel, *G*″ becomes higher than *G*′, which means that the hydrogel turns to a fluid state. When the applied strain turned to 1%, *G*′ recovered back immediately to around 3 Pa and tan δ < 1, suggesting a rapid self-healing of the hydrogel. At a second strain cycle, the hydrogel again showed self-healing after fluidization and *G*′ again subsequently recovered to the initial value. In addition, we demonstrated the self-healing ability of the hydrogel by a macroscopic self-healing test (Figure 2d). Two cube-shaped Dex **P-A** hydrogels (4 × 10 × 10 mm) were colored yellow and red by fluorescein and rhodamine B dyes, respectively. The two hydrogels were then weakly pressed together and kept in a humid atmosphere to allow self-healing. After 10 min, the two hydrogels had connected and the resulting gel could be lifted using a tweezer, without the newly formed connection failing. The crack between the two gels subsequently disappeared, enabling observable diffusion of dyes across the interface (Figure 2d). We demonstrated injection of the hydrogel by extruding a colored Dex **P-A** hydrogel through a 20G syringe needle (Figure 2e). After exiting the needle, the gel healed immediately and could be drawn as an ink.

### Cytotoxicity

**Figure 3:**
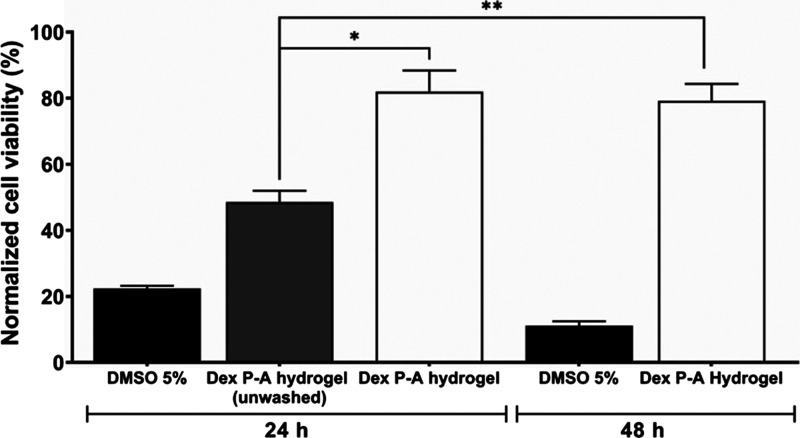
Cell viability on the Dex **P-A** hydrogel. MTT assay in D1 cells cultured on the Dex **P-A** hydrogel incubated for 24 and 48 h at 37 °C, 5% CO_2_, and 95% humidity. Error bars represent the standard error of the mean. Data correspond to three independent experiments. The asterisks indicate the difference between the unwashed Dex **P-A** hydrogel and the other hydrogels. *p = 0.01, **p = 0.007.

We next determined the cytotoxicity of the Dex **P-A** hydrogel to immune cells. For this, the dendritic cell line D1^28^ was cultured on the Dex **P-A** hydrogel, and the viability was evaluated through an MTT assay. This assay measures the reduction of MTT into formazan by mitochondrial succinate dehydrogenase.^29^ The concentration of formazan is directly proportional to the number of live cells; therefore, possible detrimental intracellular effects on metabolic activity associated with hydrogel toxicity will influence the outcome. The D1 cells were cultured on Dex **P-A** for 24 and 48 h prior to washing off the hydrogel with PBS to remove excess free reagent and any soluble byproduct. Under these conditions, 82 ± 11 and 79 ± 9% of D1 cells survived after 24 and 48 h of incubation, respectively (Figure 3). If the washing step was omitted, viability decreased by approximately 30% (*p* < 0.05 compared with 24 and 48 h incubation times), suggesting residual soluble components of the gel formation being toxic to the cells.

### Peptide Release and T-Cell Activation

**Figure 4:**
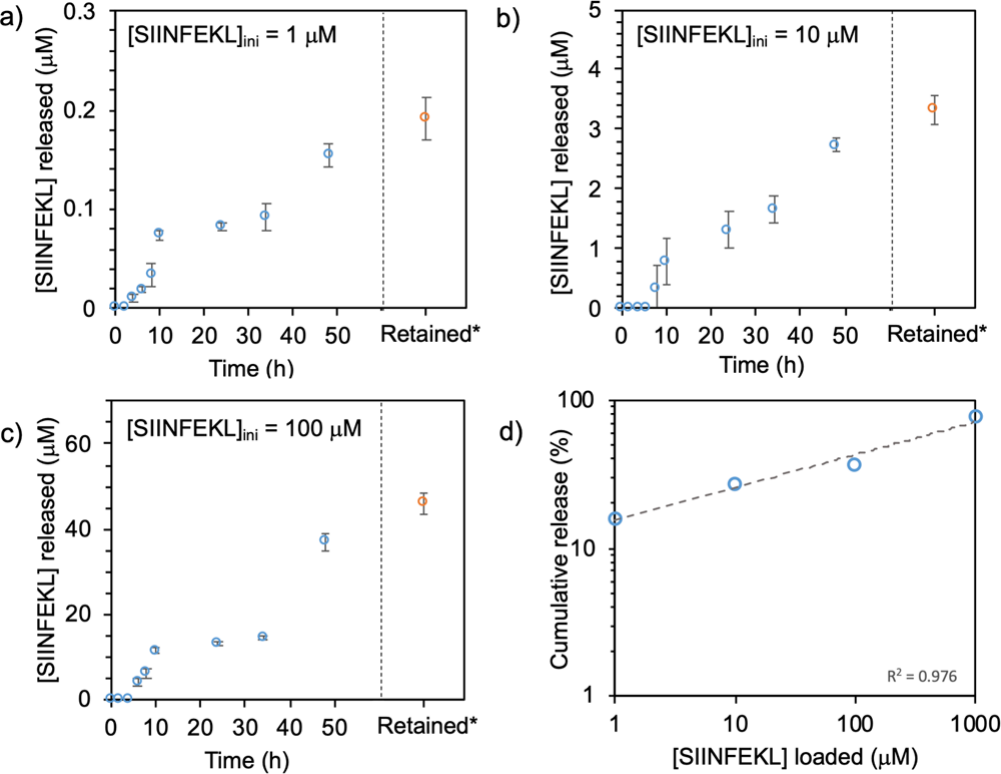
SIINFEKL release from the hydrogel. SIINFEKL release from the Dex **P-A** hydrogel over a 48 h period. D1 cells were pulsed for 3 h with the supernatant released from the Dex **P-A** hydrogel loaded with 1 μM (a), 10 μM (b), and 100 μM (c) of SIINFEKL and then co-cultured with B3Z cells to analyze their activation. *indicates SIINFEKL retained in the remaining hydrogel after 48 h. (d) SIINFEKL release profile at 48 h of Dex **P-A** hydrogels loaded with 1, 10, 100, and 1000 μM concentrations, plotted on a double logarithmic scale. The data fits a power law with a 0.23 ± 0.02 slope, describing the relation between loaded and released peptide. Dots represent the means and whiskers the SD. Data correspond to four independent experiments (*n* = 2 replicates per experiment).

To assess the potential of these hydrogels as a peptide vaccine delivery system, we evaluated the release of the commonly used minimal CD8-restricted T-cell epitope peptide SIINFEKL^30^ from the Dex **P-A** hydrogel. For this, Dex **P-A** hydrogels were loaded with three different concentrations of SIINFEKL (1, 10, and 100 μM). The release of SIINFEKL over time was detected through an (in vitro) T-cell activation assay, where the amount of peptide is quantified using the T-cell clone B3Z that carries a LacZ gene under the NFAT promoter, which allows it to produce beta-galactosidase in response to the peptide loaded on MHC-I in a concentration-dependent manner.^31^ This in turn can be quantified using the conversion of the luminogenic substrate chlorophenol red-β-d-galactopyranoside (CPRG). Figure 4 shows that the release rate of the SIINFEKL from the Dex **P-A** hydrogels is influenced by the concentration of peptide loaded in the hydrogels. In this regard, the maximum cumulative % of SIINFEKL release at 48 h is 15, 27, and 37% for the Dex **P-A** hydrogels loaded with 1, 10, and 100 μM of SIINFEKL, respectively (Figure 4a–c). An experiment at the higher 1000 μM concentration (Figure S8) confirmed this trend.

Based on the above, we compared the release profiles of SIINFEKL at 48 h from Dex **P-A** hydrogels loaded with 1, 10, 100, and 1000 μM concentrations (Figure 4d). The total amount of SIINFEKL released is related to the loading concentration by a power law with a 0.23 exponent. Combined, these results suggest that the loaded peptide is entrapped within the mesh network of the Dex **P-A** hydrogel and released either as degradation of the hydrogel matrix occurs or though diffusion from the intact matrix. The time-dependent release data (Figure 4a–c) does not show zeroth- or first-order kinetics and has only a partial fit to the Higuchi equation,^32^ suggesting that release is not merely governed by Fickian diffusion from the hydrogel matrix. A time lag observed in all release profiles also suggests that changes to the hydrogel network play a role in the release. A fit to the Korsmeyer–Peppas model^33^ shows that, after the lag time, the release process is initially largely dominated by Fickian diffusion (exponent *n* ∼ 0.5) followed by an increase of *n* on longer time scales, suggesting a combination of diffusion and hydrogel erosion. We therefore postulate that both diffusion and degradation play a role in the release, with relative contributions changing over time. Interestingly, only the gel with the extreme 1000 μM loading showed a burst release. No burst phase release of the peptide antigen was observed at the more relevant 1–100 μM loading concentrations.

The cumulative percentage of SIINFEKL release at 48 h indicates that significant amounts of SIINFEKL are retained in the hydrogel. We therefore measured the residual T-cell activation capacity of the 48 h gels. For this purpose, we disrupted the remaining hydrogel mechanically and incubated the residue at 37 °C for 20 min. After this time, the gel had dissolved completely, confirming its biodegradation potential. We subsequently pulsed D1 cells with the dissolved gels and measured T-cell activation. Interestingly, the Dex **P-A** hydrogel by itself was also able to activate the B3Z T-cells. This result suggests that the Dex **P-A** hydrogel in itself has immunostimulatory properties. This could be beneficial for increasing antigen-presenting cell (APC) activation, as has been reported in previous studies with other hydrogels and protein-dextran conjugates.^2,34^ This T-cell activation induced by non-loaded hydrogels (Figure S8) hints toward a potential local inflammatory niche formation capacity, which could enhance immune responses. However, the impact of their specific niche properties, its persistence, and relevance to the immune response induced must be explored in future in vivo assays.

**Figure 5:**
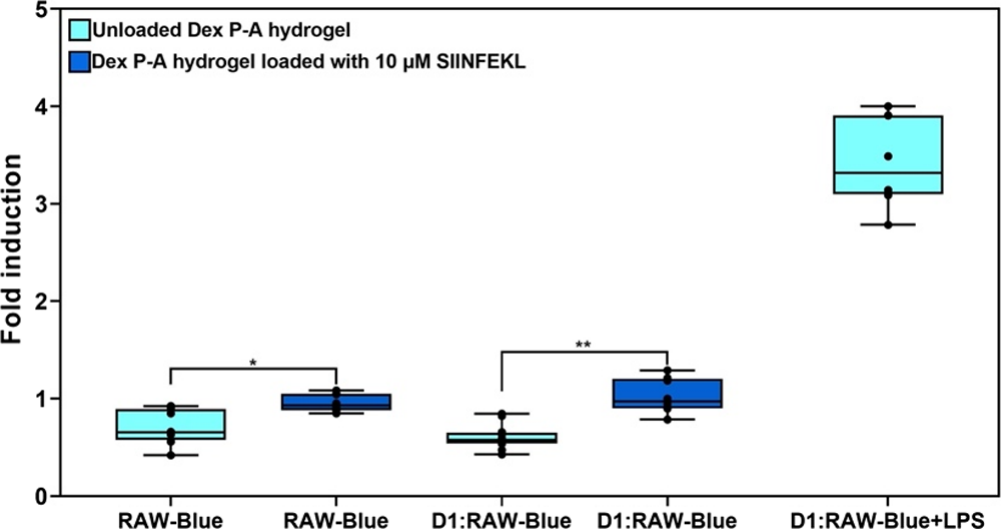
Evaluation of the activation of NF-κB/AP-1 signaling pathways in RAW-Blue cells induced by the Dex P-A hydrogel. Bars represent the mean of normalized values and error bars the standard deviation. Data were normalized using the average of SEAP release by RAW-Blue cells cultured without the Dex **P-A** hydrogel. LPS (lipopolysaccharide, a ligand of TLR4) was used as a positive control of activation of the RAW cells cultured in unloaded Dex **P-A** hydrogel. Two independent experiments were conducted. *indicates *p*-value <0.05 between RAW-Blue cells cultured in unloaded Dex **P-A** hydrogel and cells cultured in the Dex **P-A** hydrogel loaded with SIINFEKL [10 μM]. **indicates *p*-value <0.05 between RAW-Blue cells cocultured with D1 cells in unloaded Dex **P-A** hydrogel and cocultures in Dex **P-A** hydrogel loaded with SIINFEKL [10 μM].

In order to further analyze the immunological properties of the Dex **P-A** hydrogel, we evaluated the activation of RAW-Blue cells when cultured alone or with D1 cells in unloaded and loaded Dex **P-A** hydrogel. This experiment allowed us to assess the activation of these APCs due to (1) recognition of DAMPs (damage-associated molecular patterns) and (2) degradation components of the Dex **P**-**A** hydrogel able to bind to PRRs (pattern recognition receptors). Upon stimulation of PRRs such as TLRs (except TLR5), RIG-I, MDA-5, NOD, and Dectin-1, RAW-Blue cells express a secreted embryonic alkaline phosphatase (SEAP) gene inducible by the NF-κB and AP-1 transcription factors. The release of SEAP was quantified using QUANTI-Blue as described in the Supporting information. As shown in Figure 5, there is no statistically significant increase in the activation of RAW-Blue cells when cocultured with D1 cells in the unloaded Dex **P-A** hydrogel compared with RAW-Blue cells cultured alone in the Dex **P**-**A** hydrogel. This suggests that the Dex **P**-**A** hydrogel does not damage the D1 cells, and therefore, these cells do not release ligands of PRRs such as DAMPs. Additionally, we observed a statistically significant increase in the activation of RAW-Blue cells when cocultured with D1 cells in Dex **P-A** hydrogels loaded with 10 μM of SIINFEKL compared to being cocultured with D1 cells in unloaded Dex **P-A** hydrogels, which indicates that the activation of RAW-Blue (Figure 5) relies on the uptake of SIINFEKL by the antigen-presenting cells.

## Conclusions

In this work, we show how dextran-derived polymer gels can act as injectable depots for sustained release of vaccines. We developed a dextran polymer that is crosslinked using a dynamic covalent double Michael addition of thiols to alkynones. Using a masked thiol prevents undesired oxidative thiol crosslinking of the matrix polymer. The obtained hydrogels are shear thinning and self-healing and can be injected through a 20G needle, forming stable gel particles immediately after extrusion. These hydrogels show acceptable viability of dendritic cells, suggesting that they are compatible for interacting with the immune system. In vitro tests show slow, sustained release of the loaded SIINFEKL minimal epitope, as measured by T-cell activation assays. The rate of T-cell activation depends on the concentration of the loaded antigen. This, combined with the observed self-adjuvating properties of the hydrogels, suggests that they could find application as injectable depots for slow and prolonged release of vaccines, which could be used to achieve augmented vaccination response.

## Methods

NMR spectra were recorded on an Agilent-400 MR DD2 (399.7 MHz for ^1^H and 100.5 MHz for ^13^C) at 298 K. The rheological measurements were performed using a rheometer (AR G2, TA instruments) equipped with a steel plate-and-plate geometry of 40 mm in diameter and equipped with a hexadecane trap. DTT, LA, and 3-butyn-2-one were purchased from Fluorochem Ltd. Dextran-500k (*M*_n_ = 500 kDa) and dextran-20k (*M*_n_ = 20 kDa) were purchased from Alfa Aesar. Dextran-70k (*M*_r_ = 70 kDa), *N*,*N*′-dicyclohexylcarbodiimide (DCC), *p*-toluenesulfonic acid monohydrate, and 4-(dimethylamino) pyridine (DMAP) were purchased from Sigma Aldrich. 4-(Dimethylamino)pyridinium 4-toluenesulfonate (DPTS) was synthesized according to the previous literature.^35^ The technical solvents were purchased from VWR, and the reagent grade solvents were purchased from Sigma Aldrich.

### Cell Lines and Culture

D1 and B3Z (OVA_257–264_-specific, H2kb-restricted CTL hybridome) cell lines were cultured in IMDM (Iscove’s modified Dulbecco’s medium) complete medium, supplemented with 10% heated-inactivated fetal calf serum (FCS), antibiotics (100 μg mL^–1^ streptomycin and 100 IU mL^–1^ penicillin), 2 mM glutamine (glutamax), and 50 mM 2-mercaptoethanol. In addition, the D1 cell medium was supplemented with 30% rGM-CSF mouse (10–20 ng mL^–1^). This growth factor was collected and filtered from the supernatant of NIH/3T3 cell cultures. The cell lines were cultured at 37 °C with 95% relative humidity and 5% CO_2_ atmosphere. Cultured cells were harvested by PBS-EDTA and washed two times with medium.

### Synthesis of LA Anhydride

LA anhydride was synthesized based on the method described by previous literature.^36^ A mixture of LA (6.00 g, 29.08 mmol) and DCC (3.60 g, 17.45 mmol) was stirred in 80 mL of anhydrous dichloromethane at room temperature under a nitrogen atmosphere. After 20 h, the product mixture was filtered to remove dicyclohexylurea, and the solvent was removed by rotary evaporation. The product was dried in an oven overnight to obtain a yellow solid (3.59 g, yield: 60%). The product was directly used in the next step without purification.

### Synthesis of LA-Functionalized Dextran P1 by Esterification with the Acid Anhydride Method

LA-functionalized dextran (Dex-**P**) was synthesized based on the method described in the literature.^37^ Note: we used dextran from Leuconostoc spp., *M*_r_ ∼ 70,000 Da, purchased from Sigma Aldrich. Dextran from other suppliers gave unsatisfactory gelation properties.

Dextran-70k (1.41 g, containing 8.69 mmol AHG), 4-(dimethylamino) pyridine (1.06 g, 8.69 mmol), and LA anhydride [1.72 g, 4.35 mmol, ratio of LA anhydride to anhydroglucosidic rings of dextran (AHG) is 0.5] were dissolved in 20 mL of anhydrous DMSO. The mixture was stirred for 48 h at 50 °C. Then, the product was precipitated in cold ethanol. The precipitation was collected by centrifugation and dissolved in water. The mixture solution was transferred to a dialysis bag (MWCO = 14 kDa) and dialyzed by distilled water for 2 days. The white solid was obtained after freeze-drying (“**P70**”, 0.47 g, yield, 34%). The degree of substitution (DS, defined as the number of attached LA rings per 100 AHG unit) is 4.2 determined by ^1^H NMR (ratio based on the integration of peak at 3.10 ppm and the integration of peaks at 4.85 and 5.19). ^1^H NMR (399.7 MHz, D_2_O) δ 5.19, 5.05, 4.85 (m, dextran anomeric protons), 3.62 (m, hydroxyl of dextran), 3.10 (m, -SS-*CH*_*2*_-CH_2_-CH), 2.37 (m, -SS-CH_2_-*CH*_*2*_-CH and -*CH*_*2*_-COO), 1.88 (m, -SS-CH_2_-*CH*_*2*_-CH), 1.63, 1.54, 1.35 (m, -*CH*_*2*_*-CH*_*2*_*-CH*_*2*_-dithiolane ring).

Dex-**P** based on dextran-500k (**P500**) and Dex-**P** based on dextran-20k (**P20**) were synthesized by the same procedure as described above.

**P70** with varying degrees of substitution (3.1 and 5.9) was obtained by using different molar ratios of LA anhydrides to AHG of dextran (0.3 and 0.8).

### Synthesis of LA-Functionalized Dextran P70d by Esterification Using DPTS as a Catalyst System

Dextran-70k (0.981 g, containing 6.05 mmol AHG), LA (0.5 g, 2.42 mmol, 0.4 equiv to AHG of Dextran), DPTS (0.107 g, 0.363 mmol, 0.15 equiv to acid), and DCC (0.75 g, 7.27 mmol) were dissolved in 30 mL of anhydrous DMSO. The mixture was stirred for 24 h at room temperature. Then, undissolved *N*,*N*′-dicyclohexylurea was removed by filtration. The solution was precipitated in cold ethanol. The precipitation was collected by centrifugation and dissolved in water. The mixture solution was transferred to a dialysis bag (MWCO = 14 kDa) and dialyzed against distilled water for 2 days. The white solid was obtained after being freeze-dried (“**P70d**”, 0.92 g, yield, 94%).

**P70d** with varying degrees of substitution (6.8 and 10.5) was obtained by using different molar ratios of LA anhydrides to AHG of dextran (0.6 and 0.7).

### Reduction Model Reaction

The aim of the small-molecule model reaction is to study the reduction of the LA dithiolane ring by DTT in PB8.2. The reaction of sodium lipoate and DTT was followed by ^1^H NMR. To a solution of sodium lipoate (5 mg, 0.02 mmol) in PB8.2 (450 μL) and 4 drops of D_2_O, a solution of DTT (3.4 mg, 0.02 mmol) in PB8.2 (500 μL) was added, and the reaction was checked after 10 min by ^1^H NMR at room temperature.

### Preparation of Dex **P-A** Hydrogels

**P70-4.2** (40 mg, containing 0.0104 mmol LA group) was dissolved in 360 μL of phosphate buffer solution (100 mM, pH = 8.2; “PB8.2”). The mixture was stirred for 15 min at room temperature to dissolve Dex-**P** completely. DTT (16 mg, 0.104 mmol) was dissolved in 200 μL of PB8.2 as DTT pre-solution. 3-Butyn-2-one **A** (8.2 μL, 0.104 mmol) was added in 200 μL of PB8.2 as alkynone **A** pre-solution. 20 μL of DTT pre-solution (0.0104 mmol) was added to the Dex-**P** solution and stirred for 10 min. Then, 20 μL of **A** pre-solution (0.0104 mmol) was added. A turbid hydrogel (40 mg polymer in 400 μL, 10 wt %) formed after 4 h in a 1.5 mL glass vial at room temperature. Gelation was checked by the vial inversion method every half an hour.

**P70** with varying DS (3.1 and 5.9), **P500-4.8**, **P20-6.4**, and **P70d** with varying DS (4.2, 6.8, and 10.5) were tested for gelation by the method described above.

### Rheological Measurements of Hydrogels

A Dex **P-A** hydrogel was prepared as described above. A 500 μL solution of pre-gel solution was positioned on the rheometer plate. Time sweep measurements were performed at fix strain (γ = 0.5%) and frequency (ω = 6.28 rad/s = 1 Hz). Frequency sweep measurements were performed from 100 to 0.01 rad/s at a fixed strain (γ = 0.5%). All frequency sweeps were measured after storage modulus (*G*′) reached the equilibrium state. All measurements were performed in the linear viscoelastic region. The modulus of hydrogels was measured under strain sweep from 1 to 1200% at a fixed frequency (ω = 6.28 rad/s). Continuous step strain measurements were measured at the fixed frequency (ω = 6.28 rad/s). Oscillatory strains were switched from 1% strain to subsequent 1200% strain with 3 min for every strain period.

### Self-Healing Test and Injection of Hydrogels

Two pieces of a cube-shaped hydrogel (4 × 10 × 10 mm) were prepared as described above colored by rhodamine B (red dye) and fluorescein (yellow dye). Then, two pieces of different color hydrogels were brought together and kept in a moist environment for 10 min. Afterward, this healed hydrogel was cut into four equal pieces using a scalpel. Then, a piece of hydrogels was put in a syringe (1 mL volume; 0.5 inner diameter) using a tweezer and syringe plunger and subsequently injected through a 20G needle using manual force.

### Cell Viability Assay

The cytotoxicity of the Dex **P-A** hydrogel was evaluated through an MTT (tetrazolium (3-(4,5-dimethylthiazol-2-yl)-2,5-diphenyltetrazolium bromide) assay as previously described.^29^ Briefly, 85 μL of the Dex **P-A** hydrogel was added per well to a 96-well tissue-culture plate. The hydrogel was washed by adding 100 μL of PBS to eluted unreacted reagents. Then, D1 cells (100,000 cells per well) were added in 150 μL of IMDM complete medium. As a negative control of toxicity, D1 cells on IMDM complete medium were used. In addition, DMSO (5%) was used as a positive control of toxicity. The cells were incubated with the Dex **P-A** hydrogel for 24 or 48 h at 37 °C, 5% CO_2_, and 95% humidity. Next, cells were spun down 3 min at 300 × *g* at room temperature. Later, the medium was removed from each well and replaced with 90 μL of IMDM complete medium. Then, 10 μL of MTT (final concentration of 0.5 mg mL^–1^ in PBS) was added to each well and incubated for 3 h at 37 °C, 5% CO_2_, and 95% humidity. The formazan was precipitated by centrifugation, the medium was removed, and 100 μL of DMSO was added to each well to dissolve the formazan crystals. The plate was incubated for 30 min at 37 °C, with 5% CO_2_, and 95% humidity. Absorbance at 570 nm was measured using a CLARIOstar plate reader. The number of surviving cells is directly proportional to the amount of the formazan product. Results were normalized between untreated cells as 100% and only media as the background signal.

### SIINFEKL Release Assay

To evaluate indirectly the release of SIINFEKL from the Dex **P-A** hydrogel, a T-cell activation assay was carried out as previously described.^31^ Briefly, 1, 10, and 100 μM of the SIINFEKL peptide were added to the Dex **P-A** hydrogel. Then, 85 μL of the hydrogel was added per well to a 96-well tissue-culture plate. The hydrogel was washed with 100 μL of PBS 1× and later incubated with 100 μL of fresh IMDM for 2, 4, 6, 8, 10, 24, 34, and 48 h at 37 °C, 5% CO_2_, and 95% humidity. The supernatant removed from the Dex **P-A** hydrogel was diluted 1:100, 1:1000, and 1:10,000 for 1, 10, and 100 μM of the SIINFEKL loaded hydrogel, respectively. The D1 cells (50,000 cells/well) were seeded in a 96-well plate and allowed to adhere to the plate for 1 h at 37 °C, 5% CO_2_, and 95% humidity. The diluted supernatant was added to the D1 cells. The D1 cells were pulsed with 100 μL of each supernatant timeslot for 3 h at 37 °C, 5% CO_2_, and 95% humidity. The cells were spun down at 300 × *g* per 5 min, the medium was removed, and 50,000 B3Z T-cells were added per well. Co-cultures were incubated overnight (15 h) at 37 °C, 5% CO_2_, and 95% humidity.

The T-cell activation was measured as beta-galactosidase-directed CPRG hydrolysis. The B3Z T-cell line carries a lacZ construct driven by NFAT; therefore, the CPRG assay has a direct correlation with IL-2 promoter activity. For the CPRG assay, 100 μL of lysis buffer was added per well and incubated for 4 h at 37 °C in the dark. The absorbance was measured at 570 nm in a CLARIOstar plate reader.

For each assay, a standard curve was performed to interpolate the concentration of SIINFEKL available to pulsed dendritic cells, and therefore, the B3Z cells were activated. In this regard, the data was interpolated into a curve of 5–0.1 nM of the SIINFEKL peptide using a linear regression.

### Statistical Analysis

Mean and standard error of mean (SEM) and standard deviation (SD) were calculated for each variable studied. Based on the normality test, the difference between groups was assessed using a parametric test (*t* test). To interpolate the standard curve, we used a logarithmic regression. In all cases, a value of *p* < 0.05 was considered statistically significant. All graphics were performed using GraphPad Prism software, version 9.00 (GraphPad Software, La Jolla, California, USA, www.graphpad.com), and the statistical analyses were performed in R Studio (Version 0.98.1091 - 2009–2014 RStudio, Inc.) using the package pracma.

### Coculture D1 Cells:RAW-Blue Cells

200 μL of the Dex **P-A** hydrogel unloaded or loaded with 10 μM SIINFEKL was added per well to a 24-well plate. 500,000 D1 cells were seeded per well in a final volume of 300 μL of DMEM medium. A transwell insert of 0.45 microns (corning ref. 354,572) was placed per well on the plate. Within the transwell insert, 100,000 RAW-Blue cells (Invivogen, raw-sp) were seeded in 200 μL of DMEM. As a positive control of activation, 1 μg mL^–1^ LPS-EK was added to the RAW-Blue cells. Additionally, we prepared cocultures of D1 and RAW-blue cells using transwell inserts without the Dex **P-A** hydrogel. After 24 h incubation at 37 °C, NF-κB/AP-1 activation upon the previously mentioned stimuli was assessed by measuring the release of secreted embryonic alkaline phosphates (SEAPs) by the RAW-Blue cells. For this purpose, 20 μL of the induced RAW-Blue cell supernatant was added to 180 μL of QUANTI-Blue solution (Invivogen rep-qbs) in a 96-well plate. The samples were incubated for 3 h at 37 °C. SEAP release was measured at 655 nm.

## Supporting Information Available

NMR data, rheology, peptide release studies, and release models (PDF)
