# Supplementary material for: Dynamic Covalent Dextran Hydrogels as Injectable, Self-Adjuvating Peptide Vaccine Depots
Source: ACS Chem Biol. 2023 Feb 17;18(3):652–9. doi: 10.1021/acschembio.2c00938 (PMC10028604; doi:10.1021/acschembio.2c00938)
Supplement: Supplementary file 1 — cb2c00938_si_001.pdf [file cb2c00938_si_001.pdf]

Supplementary Information for

Dynamic Covalent Dextran Hydrogels as Injectable, Self-adjuvating Peptide Vaccine Depots

Bowen Fan[1,2#], Diana Torres García[3,#], Marziye Salehi[1,3], Matthew J. Webber[2], Sander I. van Kasteren[3,*], Rienk Eelkema[1,*]

[1] Department of Chemical Engineering, Delft University of Technology
Van der Maasweg 9, 2629 HZ Delft, The Netherlands

[2] Department of Chemical & Biomolecular Engineering, University of Notre Dame

Notre Dame, Indiana 46556, United States

[3] Leiden Institute of Chemistry and Institute of Chemical Immunology, Division of Bio-Organic Synthesis, Leiden University, Gorlaeus Laboratory, Einsteinweg 55, 2333 CC, Leiden, The Netherlands

# equal contributions
* corresponding authors; e-mail: r.eelkema@tudelft.nl, s.i.van.kasteren@chem.leidenuniv.nl

1. Materials Characterization

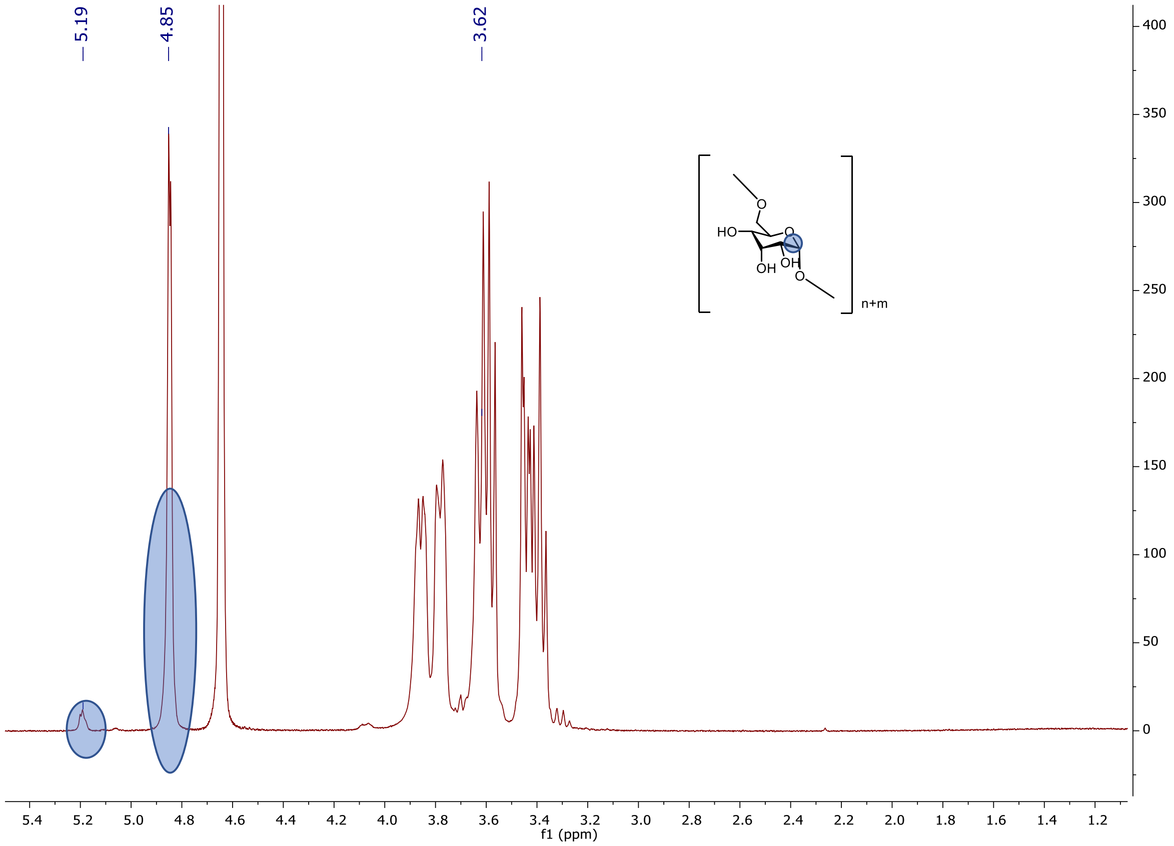

Figure S1. ^{1}H NMR spectra (399.7 MHz) of Dex70k in D_2O.

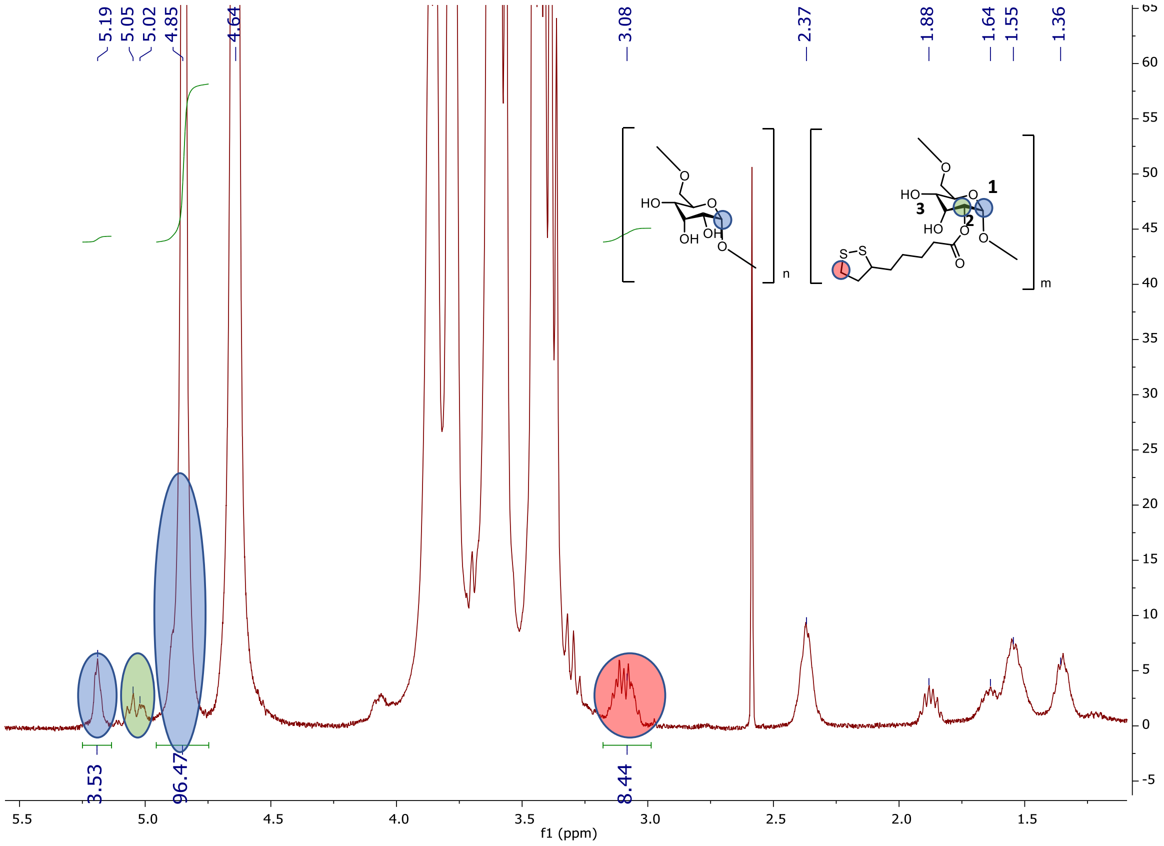

Figure S2. ^{1}H NMR spectra (399.7 MHz) of **P70-4.2** (DS 4.2) in D_2O. Blue area shows dextran anomeric protons and red area shows the protons in lipoic acid group.

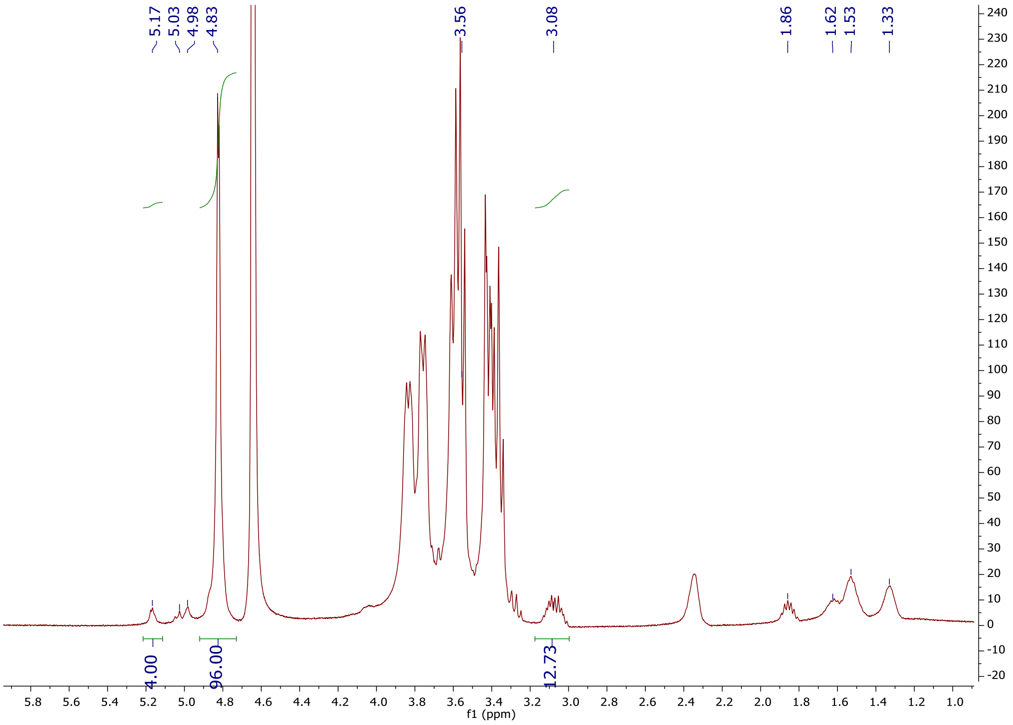

Figure S3. ^{1}H NMR spectra (399.7 MHz) of **P20-6.4** (DS 6.4) in D_2O.

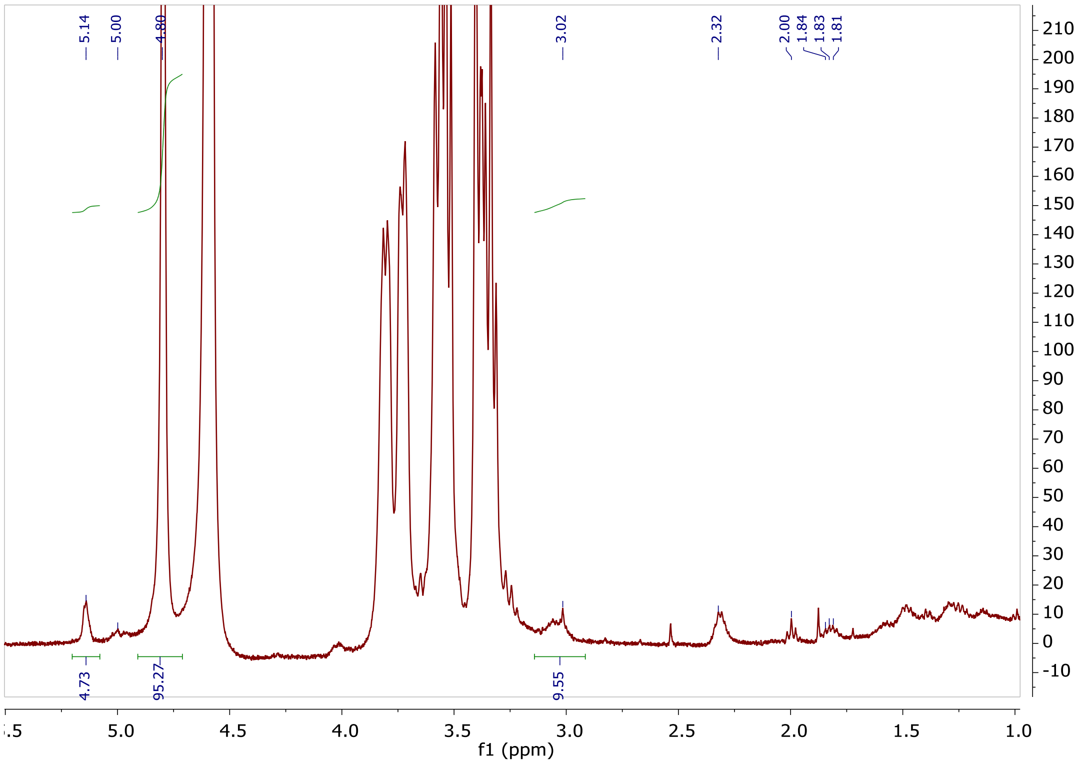

Figure S4. ^{1}H NMR spectra (399.7 MHz) of **P500-5.8** (DS 4.8) in D_2O.

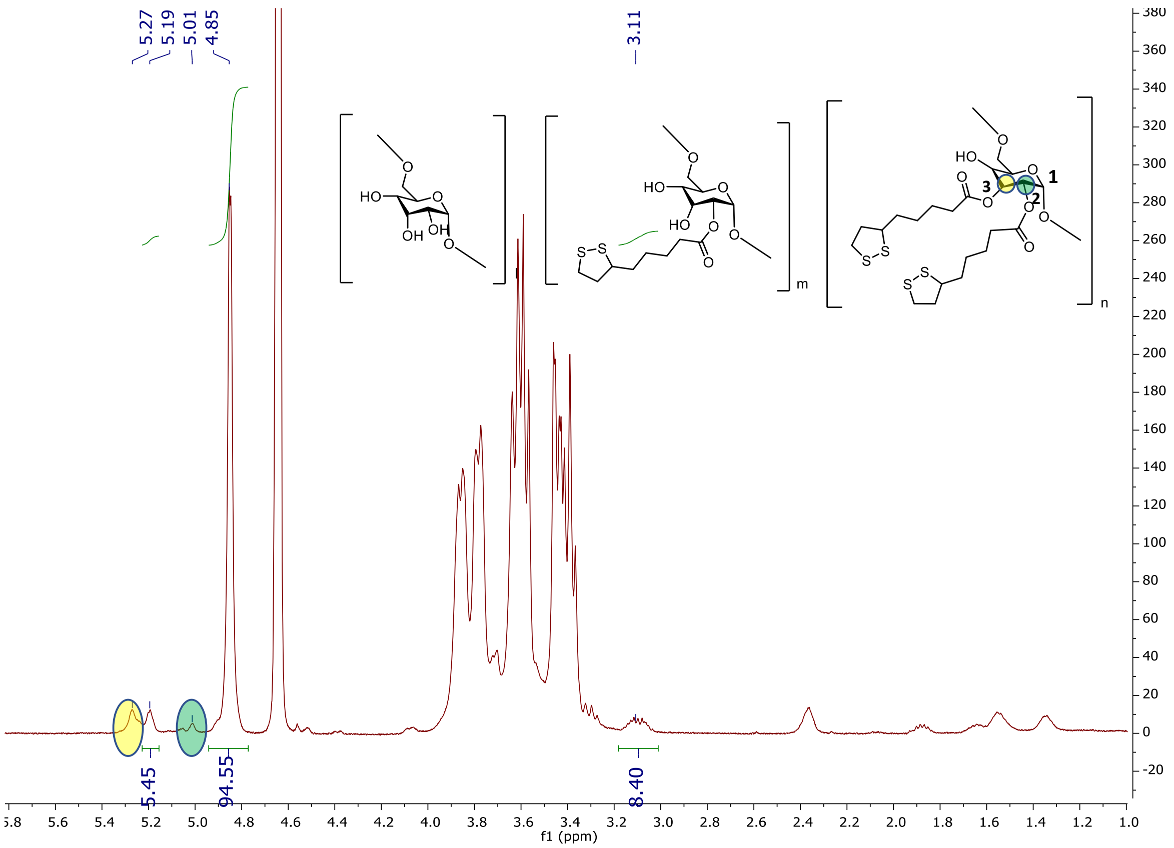

Figure S5. ^{1}H NMR spectra (399.7 MHz) of **P70d-4.2** (DS 4.2) in D_2O.

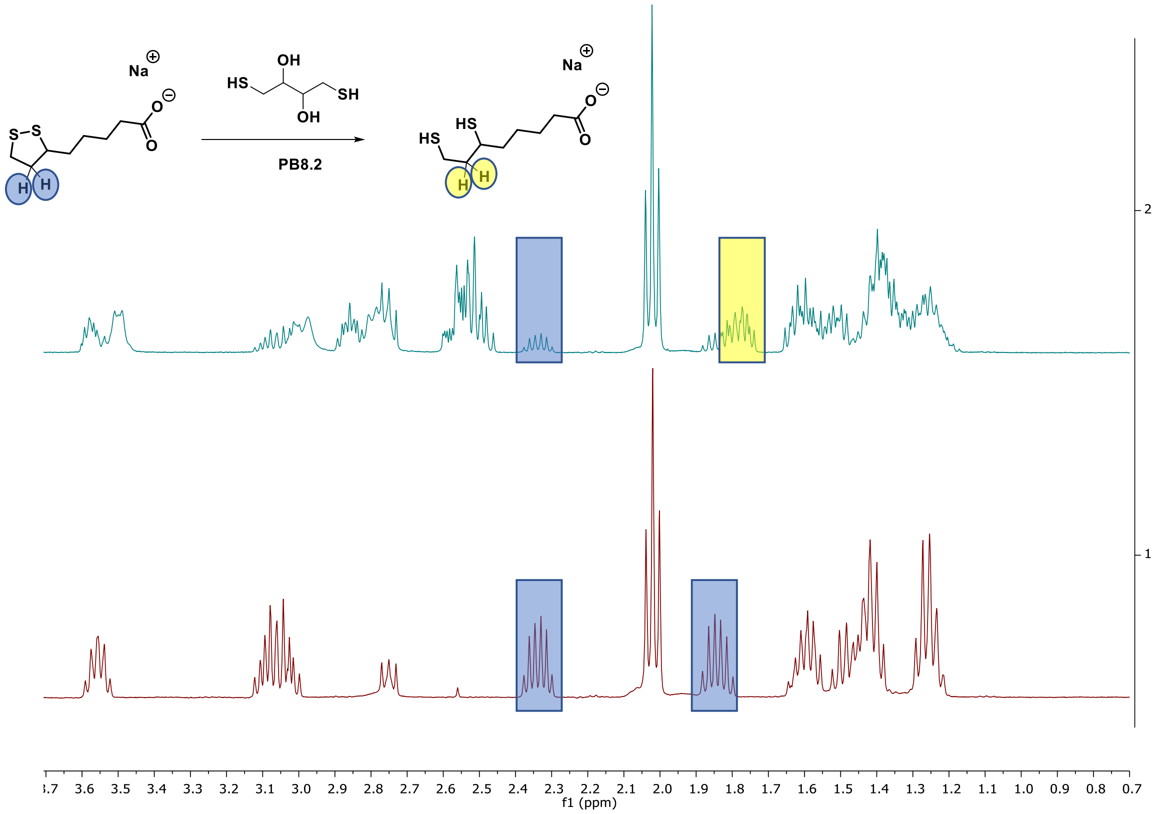

Figure S6. ^{1}H NMR (399.7 MHz, in D_2O/ PB8.2, 4 drops of D_2O in 950 μL PB8.2) monitoring of reduction of sodium lipoate by DTT at room temperature. Spectrum 1 (bottom) shows the sodium lipoate at 0 minutes and spectrum 2 (top) shows the result 10 minutes after adding DTT. The reaction can be monitored by characteristic protons: the peaks in blue square represent two protons of sodium lipoate, the peaks in yellow square represent two protons of reduced sodium lipoate.

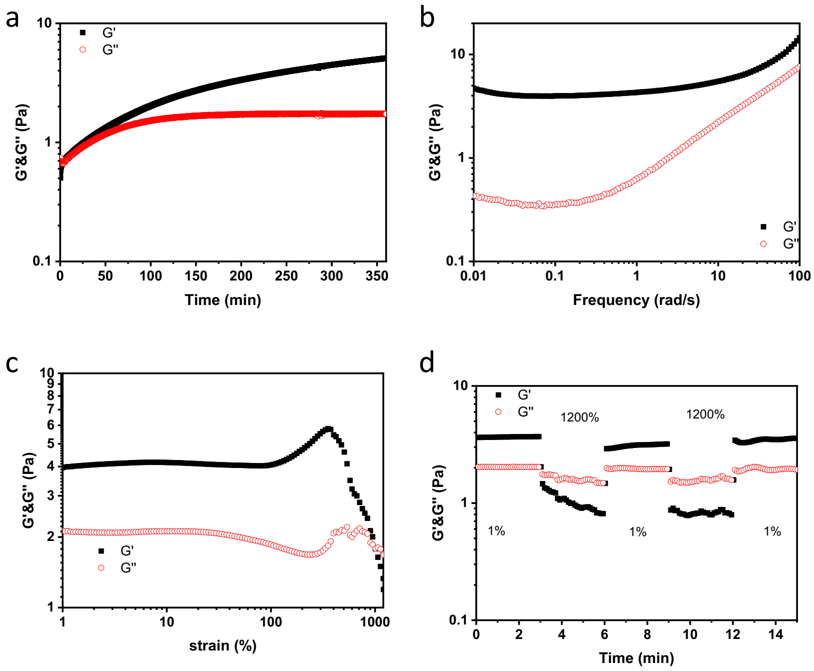

Figure S7. Rheological sweep measurements of Dex **P-A** hydrogel. a) Time sweep measurements (γ = 0.5 %, ω = 1 Hz); b) Frequency sweep measurement (γ = 0.5 %, ω = 100-0.01 rad/s); c) Strain sweep measurements (γ = 1-1200 %, ω = 1 Hz); d) Step-strain sweep, alternative strain switched from 1% to 1200% twice then back to 1%.

2. Biological Experiments

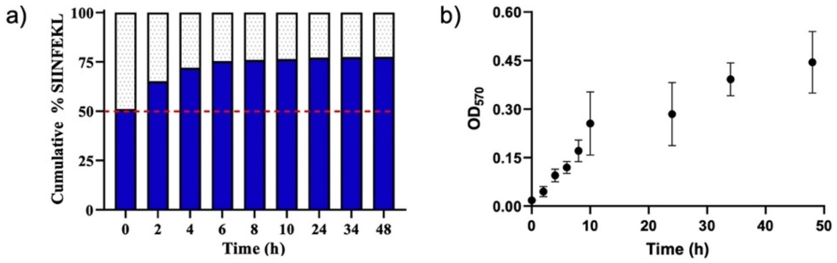

Figure S8. a) Cumulative percentage SIINFEKL release from Dex **P-A** hydrogels loaded with 1000 µM SIINFEKL. b) T-cell activation induced by non-loaded Dex **P-A** hydrogels, measured as beta-galactosidase-directed CPRG hydrolysis. Data represent three independent experiments. Error bars are the standard error from the mean.

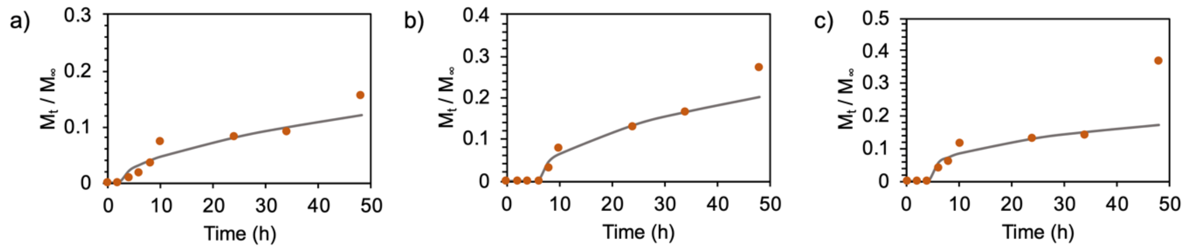

Figure S9. Fits of the Korsmeyer-Peppas model to the release data at a) 1 µM, b) 10 µM, c) 100 µM loaded peptide. We use the time-lag modified Korsmeyer-Peppas equation described below. As time-lag t_{lag} we chose the latest time point where $[\text{peptide}]_{released} = 0$. The equation was fitted by least squares optimization in MS Excel. We excluded the t = 48 h data points from the fitting optimization. Fit parameters are: a) $k = 0.015\ h^{-n}$; n = 0.55; t_{lag} = 2 h; b) k = 0.033 h^{-n}; n = 0.49; t_{lag} = 6 h; c) $k = 0.045\ h^{-n}$; n = 0.35; t_{lag} = 4 h.

$$\frac{M_t}{M_\infty} = k(t - t_{lag})^n$$

3. Release of OVA$_{323\text{-}339}$

In addition to the SIINFEKL peptide, we tested the release of OVA$_{323\text{-}339}$ from Dex **P-A** hydrogels (Figure S10). The more hydrophilic nature of the OVA$_{323\text{-}339}$ peptide resulted in an early burst release after washing, followed by gradual release plateauing after 8 hours. On long time scales (>30 hrs), a slight reduction in T-cell activation was observed. This effect may be caused by gel breakdown products impinging on MHC-II restricted antigen processing and T-cell activation.

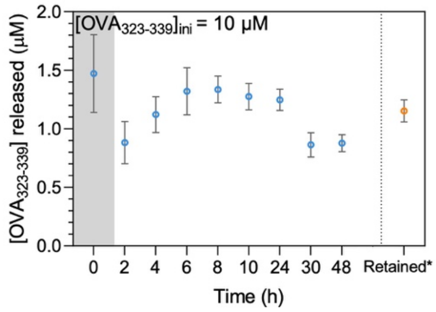

Figure S10. OVA$_{323\text{-}339}$ release from Dex P-A hydrogel. OVA$_{323\text{-}339}$ release from Dex **P-A** hydrogel over a 48 h period. A20 cells were pulsed for 4 h with supernatant released from Dex **P-A** hydrogel loaded with 10 µM of **OVA$_{323\text{-}339}$**, and then co-cultured with DO11.10 cells to analyze their activation. *Retained indicates OVA$_{323\text{-}339}$ retained in the remaining hydrogel after 48 h. Dots represent the mean and whiskers the SD. Data correspond to 3 independent experiments (n=2 replicates per experiment).

$OVA_{323\text{-}339}$ release assay: The indirect release of $OVA_{323\text{-}339}$ from the Dex P-A hydrogel was evaluated through a T-cell activation assay. Briefly, 100 µL of Dex P-A hydrogel loaded with 10 µM of $OVA_{323\text{-}339}$ peptide was added per well to a 96-well plate. After washing the Dex P-A hydrogel with PBS 1X, 100 µL of RPMI medium was added per well. The supernatant was removed after 2, 4, 6, 8, 10, and 24 h of incubation at 37 °C, 5% CO_2, and 95% humidity. Afterward, the A20 B cell lymphoma line presenting I-Ad MHC was seeded in a 96-well plate (50 000 cells/well) and pulsed for 4 h with 100 µL of each supernatant (1:10 diluted). Then cells were spun down at 300xg per 5 min, the medium was removed, and 50 000 DO11.10 T-cells were added per well. The co-cultures were incubated overnight (15h) at 37 °C, 5% CO_2, and 95% humidity. The supernatants were collected.

The T-cell activation was measured as IL-2 release. The IL-2 concentration in the supernatant was quantified by ELISA following the manufacture protocol (Invitrogen Catalog # 88-7024-88). A standard curve of 0.1-0.8 µM of OVA323-339 peptide was performed to interpolate the concentration of OVA323-339 available in the supernatants and able to activate the DO11.10 T-cells. All experiments were performed three times with two replicates each. Data were analyzed with Graphpad Prism 9 (La Jolla, CA).
